# Botanical Description of British Plants

**Published:** 1804-09-01

**Authors:** 


*August* 6, 1804.

---

### *Botanical Description of British Plants.*

( Continued from our last, pp. 124—136. )

19. Chironia. *C. centaurium. Centaurium. Gentiana centaurium.*

*Ang.* Centory. Lesser centory, or centaury.

*Gen. Desc.* Bloss. funnel-shaped, pistil leaning; stam. fixed to the top of the tube of the blossom; anthers becoming spiral; seed vessel two-celled.

*Spec. Desc.* Herbaceous, ten or twelve inches high, upright. *Leaves* spear-shaped. *Cal.* shorter than the tube, adhering to it, and that to the germen. *Flowers* a corymbus;

---

bus. *Blossom* funnel-shaped; tube long, yellowish; border reddish, segments lapping over each other. *Anthers* twisted spirally after discharging the pollen. *Style* cylindrical, cloven at the top. *Summits* two, horse-shoe shape, yellowish green. *Barren pastures, woods.* Bloss. June, August.

*Use.* Centory is justly esteemed to be the most efficacious bitter of all the medicinal plants indigenous to this country. It has been recommended as a substitute for gentian, and by some thought to be a more useful medicine; experience proves it to possess an equal degree of antiseptic power, and to it may therefore be ascribed all the medical effects of the other, viz. as a bitter tonic and stomachic, and also anthelmintic, antiseptic, emmenagogue, antiarthritic, and febrifuge. Many dyspeptic complaints are more effectually relieved by bitters than by Peruvian bark, which has now superseded the use of this and other bitters formerly common in febrile disorders; too generally perhaps, for cases of fever occur which are found to be aggravated by cinchona, yet readily yield to simple bitters. The tops, which are directed for use by the colleges, are most commonly given in infusion; but they may be taken in powder, or prepared into an extract.—*Woodville.* A tincture of the leaves, and the upper part of the root, is a good medicine in weak stomachs and cachectic habits. This plant is the basis of the famous Portland powder, which, taken in a large quantity, and for a long time together, prevents a fit of the gout, but brings on a hardness of the liver, palsy, and apoplexy. A decoction of the whole plant destroys lice, and cures the itch.—*Withering.*

20. Rhamnus. *R. catharticus. R. solutivus. Spina cervina.*

*Ang.* Buckthorn. Purging buckthorn.

*Gen. Desc.* Cal. tubular; scales protecting the stamens. Bloss. 0. Fruit, a drupa.

*Spec. Desc.* *Stem* upright, thorns terminating. *Leaves* egg-shaped. *Flowers* four-cleft, male and female, on different plants. *Blossom* pale green. *Stamens* 4. *Summit* four-cleft. *Berry* four-seeded, black. *Woods, hedges, brook-sides.* Bloss. April, May.

*Use.* The berries contain a pulpy deep-green juice, which has a bitterish, acrid, nauseous taste; they operate as a brisk *cathartic,* but their purgative effects are constantly accompanied by considerable thirst, with a dryness of the mouth and throat, and frequently with severe griping gripings of the bowels, especially unless some diluting liquor be drank immediately after taking them. The dose is said to be 20 fresh berries in substance; twice or thrice that number in decoction; one, or one and a half drachm of the dried berries; one ounce of the expressed juice, or half an ounce of the rob or extract prepared by inspissating the juice. The inner bark is said to be a strong cathartic and to excite vomiting.—*Woodville.* A purgative syrup prepared from the berries is kept in the shops, of which about an ounce is a moderate dose (2 oz. *Lightfoot*), but on account of the sickness and griping occasioned by it, is falling into disuse—*Withering.* The flesh of those birds which feed upon these berries is reported to be purgative—*Homberg. Mem de l'Ac. des Sc.* The juice of the unripe berries is of the colour of saffron, and is used for staining maps or paper: these are sold under the name of French berries. That of the ripe berries, mixed with alum, is the sap green of the painters: but if they be gathered late in Autumn their juice becomes purple, and is thus also used for a dye. The bark affords a beautiful yellow dye.—*Lin. With.* Horses, sheep, and goats eat it; cows refuse it.

21. Rhamnus. *R. frangula. Alnus nigra baccifera.*

*Ang.* Black alder, black berry-bearing alder; alder buckthorn.

*Gen. Desc.* *As above.*

*Spec. Desc.* Without thorns. *Flowers* hermaphr. one pistil. *Leaves* very entire. Bloss. five-cleft. *Summit* notched. *Berry* four-seeds, sometimes two. Inner bark, yellow; outer, sea-green; middle, blood red. *Woods, wet hedges.* Bloss. April, May.

*Use.* The inner bark, from a quarter to half an ounce, boiled in small beer, is a sharp purge. In dropsies, or constipations of the bowels of cattle, it is a very certain purgative. The berries gathered before they are ripe, dye wool of a green colour. The bark dies yellow, and, with preparations of iron, black. The wood of this shrub is preferred for charcoal used in the manufacture of gunpowder. The flowers are particularly grateful to bees. Goats devour the leaves voraciously, and sheep will eat them.—*Withering.*

22. Euonymus. *E. europæus.*

*Ang.* Spindle-tree. Prickwood. Prick timber. Gatteridge-tree. Louse berry.

*Gen. Desc.* Bloss. five-pet. caps. coloured, five-sided, five-celled, five-valved Seed coat hollow; seed veiled.

*Spec.*

*Spec. Desc.* *Flowers* mostly four cleft, greenish white. *Filaments* fixed in holes in the receptacle. *Fruit* angular, purplish red, sometimes white. *Fruit stalks* from the bosom of the leaves, with one or two pairs of flowers. *Leaves* sitting, egg-spear-shaped, opposite. *Woods, hedges.* Bloss. May, June.

In Cornwall it has four stamens.—*Mr. Stackhouse.*

*Use.* The berries act violently as an *emetic* and *cathartic.* They are *fatal to sheep.* Powdered and sprinkled upon the hair, they destroy lice. The wood, if cut when the plant is in blossom, is tough; and not easily broken; it is used by watchmakers for cleaning watches; and is made into skewers and tooth-picks. Cows are so fond of the shoots in spring, as constantly to break down the ditches of the fields wherever a plant of it stands. Goats and sheep eat it; horses refuse it.—*Withering.*

23. Viola. *V. odorata.*

*Ang.* Violet. Sweet violet.

*Gen. Desc.* Cal. five-leaved, adhering to the blossom above the base. Bloss. five pet. irregular, with a spur behind; anthers cohering, caps. one-celled, three valved.

*Spec. Desc.* *Stemless.* *Leaves* heart-shaped, suckers creeping. *Leaf-stalks* nearly smooth. *Fruit-stalks* channelled on the upper side. *Flowers* with or without petals, producing perfect seed. *Bloss.* purple, blue, or white; very fragrant. *Warm hedges, moist warm lanes, in clay or marle.* Bloss. March, April.

*Use.* The violet was used by the antients in various inflammatory diseases, and was alluded to by the poets as a vulnerary. *Ovid Met.* The recent flowers only are now admitted into the Materia Medica; they have an agreeable smell, and a mucilaginous bitter taste; to water they readily give out their virtue and flavour, but with difficulty to rectified spirit. Taken in the quantity of a drachm or two they are gently *purgative*; and, according to Bergius and some others, possess an anodyne and pectoral quality. The officinal preparation of them is a syrup, which to young children answers the purpose of an agreeable purgative. The seeds are said to be strongly *diuretic,* and useful in gravelly complaints; and the root powdered, in a dose of a drachm, proves both *emetic* and *cathartic.*—*Woodville.* Both the flowers and seeds are said to be mild laxatives.—*Withering.* The flowers, and also the leaves, are cooling and emollient.—*Lightfoot.* The syrup of violets, which receives its colour from the petals, is very useful in chemical enquiries, to detect an acid or an alkali; the the former changing the blue colour to a red, and the latter to a green: for this purpose the violet is cultivated in large quantities about Stratford upon Avon. Slips of white paper, stained with the juice of the petals, and kept from the air and light, answer the same purpose.—*Withering.* The Turks make a violet-sugar of the flowers, and this dissolved in water constitutes their favourite liquor called Sorbet.—*Lightfoot.*

24. Viola. *V. tricolor.*

*Ang.* Pansies. Heart's ease. Three faces under a hood. Love in idleness. Kiss at the garden gate. Call me to you. Herb Trinity.

*Gen. Desc.* *As above.*

*Spec. Desc.* With a stem, stipulæ wing-cleft, terminating at the end of the leaf stalk; summit urn-shaped. *Stem* branched, weak. *Leaves* egg-shaped, toothed. *Floral leaves* two on each fruit stalk, halberd-shaped. *Calyx* smooth, half the size of the blossom. *Summit* hollow, open, fringed on the lower part. *Bloss.* subject to great variety of colour, white, yellow, blue, purple, two or more of these. *Corn fields, ditch-banks, &c.* Bloss. May, September.

*Use.* Dr. Strack says, that this plant infallibly cures the *scabby* complaints in young children, called *crusta lactea.* He boils a handful of the fresh, or half a *drachm* of the dried leaves, in half a pint of milk; and gives this milk, strained, morning and evening, for some weeks.—*Medical Journal,* ii. p. 188. By many of the old writers on Materia Medica, this plant is represented as a powerful medicine in *epilepsy, asthma, ulcers, scabies,* and *cutaneous* complaints; but by modern authorities it has been recommended chiefly as a remedy for the crusta lactea. In addition to the dose above mentioned, bread, with this decoction, is also to be formed into a poultice and applied to the part: by this treatment it has been observed that the eruption during the first eight days increases, and that the urine, when the medicine succeeds, has an odor similar to that of cats; but on continuing the use of the plant a sufficient time, this smell goes off, the scabs disappear, and the skin recovers its natural purity. Haase administered this violet in various forms and in large doses, extending its use to many chronic diseases, *(see H. de viola tricolore, Erlang.* 1782.) and from the numerous instances of its success, it seems well deserving of further trial.—*Woodville.*

25. IMPATIENS. *J. Noli-tangere.*

*Ang.* Quick in hand. Impatient. Touch me not.

*Gen. Desc.* Cal. two-leaved. Bloss. five petal, irregular. Nectary hoodlike. Stem, cohering; caps, superior, one-celled, opening with a jerk into five spiral valves.

*Spec. Desc. Fruit stalks* many fl. solitary. *Leaves* egg-shaped, pendant at night. *Stem;* swoln at joints. *Bloss.* yellow with red spots. *Moist, shady places.* BLOSS. July, August.

*Use.* The whole plant is considerably acrid. Goats feed upon it; horses, cows, and sheep, refuse it.—*Withering.*

26. RIBES. *R. rubrum.*

*Ang.* Currant. Red currant.

*Gen. Desc.* Petals five, they and the stamens fixed to the calyx. Style cloven. Berry beneath, one-celled, many-seeded.

*Spec. Desc. Without prickles. Bunches* smooth, pendant; flowers, flattish; *leaves,* segm. rounded; cal. spreading. *Bloss.* greenish-white. *Berries* red, sometimes white. *In woods in the northern counties, banks of the Tees.* BLOSS. May.

*Use.* The fruit of this plant is universally acceptable, either as Nature presents it, or variously prepared by art with the addition of sugar. Its medicinal qualities are similar to those of other subacid fruits, which are esteemed to be moderately *refrigerant, antiseptic, attenuant,* and *aperient.* Hoffman and Boerhaave had great confidence in the efficacy of these fruits in obstinate visceral obstructions. They may be used with advantage to allay thirst in most febrile complaints; to lessen an increased secretion of bile; and to correct a putrid and scorbutic state of the fluids, especially in sanguine constitutions; in those of a contrary kind, they are apt to occasion flatulency and indigestion. The juice of the red currant is a most agreeable acid in punch; and at Paris, mixed with sugar, it is a common beverage, generally preferred to orgeat or lemonade.—*Woodville.* Cows, sheep, and goats eat the leaves; horses are not fond of them.—*Linn.*

27. RIBES. *R. nigrum. Grossularia non spinosa fructu nigro.*

*Ang.* Black currant. Squinancy berries.

*Gen. Desc. As above.*

*Spec. Desc. Bunches* hairy; *flowers* oblong, woolly; *floral leaves* woolly, as long as the little fruit-stalks. *Buds* glandular. Cal. segm. of a rich brown colour. *Petals* sometimes sometimes change into stamens. *Wet hedges, river banks.* Bloss. May.

*Use.* The berries have a very peculiar flavour, which many people dislike; but, together with the properties which they possess in common with other subacid fruits, they are peculiarly useful in sore throats, especially those of the inflammatory kind: for this purpose the juice is boiled down to an extract with the addition of a small proportion of sugar, in that state called *rob.* There is little doubt that, in cases of inflammatory *angina,* they may be advantageously used to answer the same intentions as gargles. They are said to possess a *diuretic* power also to a considerable degree, but this seems to want confirmation.

The common black currant jelly in domestic use, for the cure of sore throats, it may be observed, loses much of its efficacy by having too great a proportion of sugar in its preparation. These berries by some people are put into brandy, for the same purpose as black cherries, and more commonly in Ireland, into whiskey. The tender leaves tinge common spirits so as to resemble brandy: the leaves are said, in an infusion, to have a taste of green tea, and when young are thus, to some, peculiarly agreeable. These leaves are extremely fragrant, and have likewise been recommended for their medicinal virtue, which Bergius states to be *mundificans, pellens, diuretica.* An infusion of the young roots is useful in fevers of the eruptive kind, and in the dysenteric fevers of cattle. Haller says, that of this fruit, a wine may be made equal to any produced from the grape.—*Woodville.*—*Withering.*—*Haller.*— Goats and horses eat the leaves.—*Linnè.*

28. Hedera. *H. helix.* *H. arboracea.*

*Ang.* Ivy. Common Ivy.

*Gen. Desc.* Petals five, oblong; berry, four or five-celled; three to five-seeded; juiceless; encircled by the calyx.

*Spec. Desc.* *Leaves,* when weak and trailing, lobed; when climbing and strong, egg-shaped; glossy. *Bloss.* greenish-white. *Berry* black. *Woods, old walls.* Bloss. October.

*Use.* The leaves have a nauseous taste. Haller observes, that they are given in Germany as a specific in the *atrophy* of children. Common people apply them to issues.—*Withering.* In Scotland the Highlanders make an ointment of the leaves, which is much valued by them as a ready cure for burns.—*Lightfoot.* The berries have a little tle acidity; they purge and vomit. In warm climates a resinous juice exudes from the stalks. The roots are used by leather-cutters to whet their knives upon. The evergreen leaves adorn our walls and cover the naked trunks of trees. Apricots and peaches, covered with ivy during the month of February, have been observed to bear fruit plentifully.—*Withering.* Horses and sheep eat it; cows and goats refuse it.—*Linnæus.* The seeds of ivy, blown by the wind from the tops of trees and houses, have sometimes been supposed to have rained from the clouds; and a distinguished member of the Royal Society declared them once to be wild garlick.—*Hill.*

Pentandria, Digynia.

29. Herniaria. *H. glabra. Polygonum minus.*

*Ang.* Smooth rupturewort.

*Gen. Desc.* Cal. five divisions. Bloss. 0; stam. five perfect, and five imperfect; caps. one-seeded.

*Spec. Desc.* Plant smooth, four to eight inches long, trailing, wood-like, knotted at the bottom; *flowers* numerous, yellowish, without petals. *Floral leaves* triangular, fringed. *Gravelly soil.* Bloss. July, August.

*Use.* A little saltish and *astringent.* It increases the secretion by the *kidnies.* The juice takes away specks in the eye. Horses, cows and sheep eat it; goats and swine refuse it.—*Withering.*

30. Chenopodium, *C. Bonus, Henricus. Lapathum unctuosum.*

*Ang.* Mercury goosefoot. Common English mercury. All good. Good Henry. Good King Henry. Wild spinage.

*Gen. Desc.* Cal. five-cleft, five-ribbed. Bloss. 0; seed, one; round, flatted, superior, horizontal, covered by the closing calyx.

*Spec. Desc.* *Leaves* triangular-arrow-shaped, entire, waved. *Spikes* compound, leafless, axillary; *little spikes* alternate sitting. *Bloss.* greenish-white; *flowers* congregated, sitting. *Rubbish, road-sides, walls.* Bloss. May.

*Use.* This plant is cultivated as spinage by the poor at Boston, in Lincolnshire.—*Curtis.* The tops are eaten boiled; and a decoction of the leaves is used in emollient clysters.—*Hill.* The young shoots, peeled and boiled, may be eaten as asparagus, which they resemble in flavour. They are gently *laxative.* The leaves are often boiled in broth. The roots are given to sheep that have a cough.

Goats and sheep eat it, but are not fond of it; horses, cows, and swine, refuse it.—*Withering.*

31. Chenopodium. *C. olidum. C. vulvaria. Blitum fœtidum.*

*Ang* Stinking orache. Stinking goosefoot.

*Gen. Desc. As above.*

*Spec. Desc. Leaves* very entire, diamond-egg-shaped; *flowers* congregated, axillary, trailing, and smell like salt-fish. *Road-sides, old walls.* Bloss. August.

*Use.* Its smell is rank and fetid; it has got the reputation of being an anti-hysteric. Horses, cows, sheep and goats eat it; swine refuse it.—*Withering.*

32. Chenopodium. *C. maritimum.*

*Ang.* Small white glasswort. Small glasswort. Sea goosefoot.

*Gen. Desc. As above.*

*Spec. Desc. Leaves* awl-shaped, accurately semicylindrical. *Branches* alternate. *Flowers* solitary, axillary. *Style* single. *Summits* three, pink-coloured. *Seeds* glossy. *Sea shore.* Bloss. August.

*Use.* This plant is an excellent pot-herb.—*Withering.*

33. Atriplex. *A. nastata.*

*Ang.* Wild orache, Fat-hen. Lamb's quarters.

*Gen. Desc.* Bloss. 0. Flowers, some hermaphrodite, some female, on the same plant; hermaphrodite cal. five-leaved. Seed 1, depressed, upright. Female, cal. two-leaved. Seed 1, compressed.

*Spec. Desc. Stem* herbaceous, upright or trailing, angular, furrowed. Calyx valves of the female flowers large, trowel-shaped, indented. *Leaves* trowel or halberd-shaped, indented, or toothed, or entire. Varies much. *Rubbish, dunghills.* Bloss. August, September.

*Use.* This plant is sometimes gathered as a pot-herb, and eaten in lieu of spinage and other greens. Cows, sheep, goats and swine eat it, but are not fond of it.—*Withering.*

34. Humulus. *H. lupulus. Lupulus mas. et fœmina.*

*Ang.* Hop.

*Gen Desc.* Male and female flowers on different plants. Bloss. 0; male cal. five-leaved; female cal. one-leaved, with a slanting opening, entire. Seed one, with a leaf-like calyx.

*Spec. Desc. Stems* climbing. *Leaves* lobed, serrated. Flowers greenish-yellow. *In hedges.* Bloss. July.

*Use.* The flowers of the female plant are the common hops hops used in brewing ale and beer; they grow wild in the hedges, but the value of the female flowers renders it worthy of being carefully cultivated: Soil and cultivation occasion some varieties, but for the purposes of brewing, they are commonly distinguished by the names of Kentish or Worcestershire hops. Hop yards might be preserved from the honey-dew, which is the excrement of a species of *aphis,* and from the ottermoth, by being covered with stones. *See Withering Bot. Ar. l. c. note.* A decoction of the young shoots is esteemed a powerful lithonthriptic. —*Lightfoot.* A bag of *hops* placed under the pillow is an excellent soporific.—*Dr. Addington.* A decoction of the roots, or from twenty to thirty grains of the extract, is said to be sudorific, and to answer the purposes of sarsaparilla. The young shoots boiled, and eaten as asparagus, early in spring, are esteemed a delicacy; they are sold under the name of *hop-tops.*—It will dye wool yellow. Strong cloth is made in Sweden from the stalks; which, for that purpose, are gathered in autumn, soaked in water all the winter, and in March, after being dried on a stove, dressed like flax. Horses, cows, sheep, goats and swine eat it. —*Withering.*

What is that electrical murmur, like very distant thunder, when hop-poles are shaken by the wind?—*Linnæus.*

35. Salsola. *S. Kali. Kali spinosum cochleatum. Tragus spinosas.*

*Ang.* Prickly glass-wort. Kelp-wort.

*Gen. Desc.* Cal. five-cleft. Bloss. 0; seed 1, beneath; coated by the calyx.

*Spec. Desc.* Herbaceous, lying down. *Leaves* awl-shaped, thorny, rough; *calyxes* bordered, axillary; *flowers* greenish. *Sandy sea shores.* Bloss. July, August.

*Use.* This, with several other species of the same genus, are among the marine plants, from which the fossil alkali, known by the name of barilla and soda ashes, is obtained. This species is most in use in Eastern countries. On the coasts of the Mediterranean, where the preparation of these ashes forms a considerable branch of commerce, the seeds of the salsola are regularly sown in a proper situation near the sea. They shoot in about a fortnight; and about seed time they are pulled up by the roots, and dried, when the seeds are collected; then tied in bundles, and burnt in an oven constructed for the purpose, where the ashes, while hot, are continually stirred with long poles. On becoming cold the saline matter forms a hard solid mass, which is afterward broken into convenient pieces.—*Woodville.*

36. Ulmus. *U. campestris. U. sylvestris.*

*Ang.* Common elm.

*Gen. Desc.* Cal. five-cleft. Bloss. 0; caps. superior, one-celled, leaf-like, compressed; seed solitary.

*Spec. Desc. Leaves* doubly serrated, unequal at the base; *flowers* almost sitting, crowded, in a kind of corymbus. *Flower-buds* beneath the leaf-buds. *Bark* cracked and wrinkled. *Hedges, hedgerows. In the South of England.* Bloss. April.

*Use.* The inner tough bark, which is directed for use by the pharmacopœias, has a bitterish taste, but no peculiar smell: it abounds with a slimy juice, recommended in *nephritic* cases, and externally as a useful application to burns: this bark, on the branches, is more bitter than on the trunk. The outer bark has neither taste nor smell, and contains but little mucilage. The complaints for which this medicine has been used are chiefly those of the cutaneous kind, allied to herpes and lepra. Dr. Lysons mentions five cases of inveterate eruptions, successfully treated by a decoction of this bark, prepared from four oz. taken fresh and boiled in two quarts of water to one; of this half a pint was given twice a day. But as he added nitre, and used frequent purgatives, it may be doubted whether the cure should wholly be ascribed to the elm bark. It was found efficacious by Dr. Lettsom in the *lepra ichthyosis* of Sauvages; and in the *lepra vulgaris*, in a remarkable instance, by Banau; who proposes the use of it in rheumatism, fluor albus, old ulcers, cancerous and scrophulous affections, tinea capitis, scurvy, &c. (*Journal de Paris,* 1783, *n.* 255.) An obstinate perseverance of some months is requisite.—*Woodville.* This decoction, drank freely, has been known to carry off the water in *dropsies.*—*Withering.* The bark, dried and ground to powder, has been mixed with meal in Norway to make bread, in times of scarcity.—The inner bark, boiled in water, makes an excellent mucilage used in sore throats and fevers.—*Hill.* The flowers have a violet smell. The wood, being hard and tough, is much used to make axle-trees, mill wheels, boat keels, chairs, coffins, &c. This tree bears transplanting, loves an open situation, and black or clayey soil, and does not destroy the grass under it: if grafted on the *U. glabra* it will not throw out suckers, with which it is apt to over-run the ground. Horses, cows, goats, sheep and swine swine are fond of the leaves.—*Withering* And the tender leaves are devoured with avidity by silk worms. *Tr. Soc. Arts. ii.* 157.

37. Xanthium. *X. strumarium. Lappa minor.*

*Ang.* Lesser burdock. Burdock clottweed.

*Gen. Desc.* Male and female fl. on the same plant. *M.* cal. common, tiled. Bloss. one-petal, funnel-shaped, five-cleft; recept. chaffy. *Fem.* involucr. two-leaved, two-flowered. Bloss. 0; caps. double, prickly, cloven; nut two-celled.

*Spec. Desc.* *Stem* thornless. *Leaves* heart-shaped, three-fibred. *M. fl.* in a branched bunch, terminating. *F. fl.* in bosoms of upper leaves, *fruit* oblong, echinated. *Dung-hills.* Bloss. June, September.

*Use.* The leaves are bitter and astringent. A decoction of the whole plant affords a bright showy yellow colour; but it is better if only the flowers are used. Horses and goats eat it; cows, sheep and swine refuse it.—*Withering.*

38. Eryngium. *E. maritimum.*

*Ang.* Sea eryngo. Sea holly.

*Gen. Desc.* Flowers forming a head; gen. invol. many-leaved; recept. chaffy; seeds rough, with flexible scales.

*Spec. Desc.* *Root leaves* roundish, plaited, thorny, three-cleft. *Upper leaves* and *leaf-stalks* embracing the stem. *Flowering heads* on fruit-stalks; chaff three-pointed. *Leaves* mealy, with a whitish wood-like border; angles ending in sharp whitish thorns. *Bloss.* whitish blue. *Sea shore.* Bloss. July, August.

*Use.* The root, the part directed for medicinal use, exhibits to the taste a grateful sweetness; and being chewed for some time, discovers a light aromatic warmth or pungency. Boerhaave esteemed this the principal of the *aperient* roots, and he usually prescribed it as a *diuretic* and *antiscorbutic.* It has likewise been celebrated for its aphrodisiac powers: but the effects of eryngo seem now to obtain but little credit. The root is frequently sold in the shops candied; and is made into a sweat-meat.—*Woodville.* The leaves are sweetish, with a light aromatic warmth and pungency.—*Withering.* The young flowering shoots boiled, have the flavour of asparagus.—*Linn.*

39. Daucus. *D. carota. D. sylvestris. D. vulgaris. D. polygamus.*

*Ang.* Wild carrot. Bird's nest.

*Gen. Desc.* Bloss. somewhat radiated; florets hermaph. leafits of the involucr. divided; seeds with membranaceous toothed ridges.

*Spec. Desc.* Angles of *seeds* 4, distant, hispid; leaf-stalks, fibrous underneath; umbel concave when in seed; flowers white or reddish. *Meadows, pastures.* Bloss. July, August.

There are several varieties of this species. *See Bot. Arrangement.*

*Use.* The seeds have a light aromatic smell, and a warm acrid taste; they possess, though not in a considerable degree, the aromatic qualities common to those of most of the umbelliferous plants, and hence they have long been deemed *carminative* and *emmenagogue;* but they are chiefly esteemed for their diuretic powers, and for their utility in *calculous* and *nephritic* complaints: in which an infusion of three spoonsfull of the seeds in a pint of boiling water has been recommended; or the seeds may be fermented in malt liquor, which receives from them an agreeable flavour, resembling that of lemon peel. Carrots have been given to children as a vermifuge. The expressed juice, or a decoction of the roots has been recommended in calculous complaints, and as a gargle for infants in *apthous* affections, or excoriations of the mouth; and a poultice of scraped carrot has been found useful, applied to *phagedenic* ulcers, and to *cancerous* and *putrid* sores; as well in mitigating the pain as in abating the smell.—This plant, in its cultivated state, is the well known garden carrot, which, as it possesses a large proportion of saccharine matter, affords consequently much nourishment, but it is found to be difficult of digestion.—*Woodville.* An infusion of the seeds in ale or water has been found to give immediate relief in sharp fits of the gravel.—*Lightfoot.*—*Withering.* The roots are a grateful and nutritious food for all kinds of cattle, and well worthy of attention; but, if given too long, they are apt to occasion bloody urine. *Ibid.*

[To be continued.]

